# Computed tomography-guided percutaneous gastrostomy: initial experience at a cancer center

**DOI:** 10.1590/0100-3984.2015.0219

**Published:** 2017

**Authors:** Chiang Jeng Tyng, Erich Frank Vater Santos, Luiz Felipe Alves Guerra, Almir Galvão Vieira Bitencourt, Paula Nicole Vieira Pinto Barbosa, Rubens Chojniak

**Affiliations:** 1 PhD, Attending Physician, Imaging Department, A.C.Camargo Cancer Center, São Paulo, SP, Brazil; 2 MD, Radiologist, Resident in Interventional Radiology, Imaging Department, A.C.Camargo Cancer Center, São Paulo, SP, Brazil; 3 MD, Resident in Radiology and Diagnostic Imaging, Hospital Universitário Cassiano Antonio de Morais – Universidade Federal do Espírito Santo (HUCAM-UFES), Vitória, ES, Brazil; 4 PhD, Head of the Imaging Department, A.C.Camargo Cancer Center, São Paulo, SP, Brazil

**Keywords:** Gastrostomy, Radiology, interventional, Tomography, X-ray computed

## Abstract

Gastrostomy is indicated for patients with conditions that do not allow adequate oral nutrition. To reduce the morbidity and costs associated with the procedure, there is a trend toward the use of percutaneous gastrostomy, guided by endoscopy, fluoroscopy, or, most recently, computed tomography. The purpose of this paper was to review the computed tomography-guided gastrostomy procedure, as well as the indications for its use and the potential complications.

## Abstract (pt)

A gastrostomia é indicada para pacientes com condições que não permitam uma alimentação adequada por via oral. A tendência atual é realizá-la por meio de técnicas percutâneas, guiadas por endoscopia, fluoroscopia ou, mais recentemente, por tomografia computadorizada, reduzindo a morbimortalidade e custos do procedimento. O objetivo deste trabalho é revisar a técnica de gastrostomia guiada por tomografia computadorizada, suas indicações e complicações.

## INTRODUCTION

The purpose of gastrostomy is to provide access for prolonged enteral nutrition in patients with acute or chronic conditions that preclude adequate oral nutrition and who therefore present a risk of developing malnutrition or other nutritional deficiencies^(1)^. In the past, gastrostomy consisted of a surgical procedure. However, the current trend is to undertake the procedure using percutaneous techniques, guided by endoscopy, fluoroscopy, or, more recently, computed tomography (CT), thus reducing morbidity, mortality, the costs of the procedure, and patient recovery time^(2)^. The most common indications for the use of gastrostomy are determined by changes in the swallowing function, secondary to central nervous system dysfunctions (benign and malignant) or resulting from obstructive neoplastic lesions of the upper aerodigestive tract (oropharynx, larynx, hypopharynx, or esophagus)^(3)^.

In terms of the success rates and complications of percutaneous procedures, there is little difference between endoscopic and radiological approaches. Due to the growth of interventional radiology and long waiting lists for endoscopic procedures, radiological approaches have gained prominence in recent years, supplanting endoscopic approaches at some centers, mainly for patients with head and neck tumors^(4-6)^. It should be borne in mind that the presence of neoplastic lesions in the upper aerodigestive tract add a potential source of difficulty, related to the endoscopic procedure, given that extensive tumor masses or infiltrative stenotic lesions can block the passage of the device to the stomach^(7,8)^. In addition, in patients developing anatomical alterations after the surgical resection of head and neck tumors and post-radiotherapy alterations, there is the further risk of obstruction of the upper airway due to edema, fibrosis, or narrowing of the glottis^(8,9)^. Patients with locally advanced tumors at the base of the tongue, larynx, or hypopharynx are at greater risk. In such patients, CT-guided gastrostomy has proven to be an efficient treatment option, given that it does not involve manipulation of the upper digestive tract, which reduces the failure rate of the procedure, as well as the risk of airway obstruction^(10)^.

In Brazil, CT-guided gastrostomy is still a new procedure, available at few facilities. The purpose of this paper was to review the literature on CT-guided gastrostomy, considering the indications for its use, the techniques employed, and the potential complications.

## INDICATIONS

Percutaneous CT-guided gastrostomy can be carried out in various clinical contexts, especially in situations unfavorable to endoscopic approaches, such as in patients who have undergone partial gastrectomy, in whom the small volume of gastric remnant hampers adequate transillumination for the endoscopic placement of the gastrostomy tube, or in patients with advanced tumors at the base of the tongue, larynx, and hypopharynx, because infiltrative, stenotic masses in the upper aerodigestive tract impede the progress of the endoscope toward the gastric lumen^(6,8)^.

Patients with advanced neoplasm of the head and neck require special attention, because they present significant deterioration of their quality of life, mainly owing to the location of these tumors, which, when invasive, can affect the swallowing function, with direct consequences on the nutritional status and survival of these individuals^(11)^. However, the difficulty in maintaining adequate ingestion of nutrition is a consequence not only of the neoplasm but also of the treatment used, which varies from extensive resection of the oropharynx to chemotherapy and radiotherapy. Other factors that contribute to nutritional deficiencies include cranial nerve lesions, adynamic pharyngeal segments, bronchial aspiration, alcoholism, smoking, an unbalanced diet, and, occasionally, the development of postoperative fistulas^(12,13)^. Strict nutritional guidance and intravenous administration of high-calorie and high-protein supplements are recommended in these cases, although they have proven to be ineffective in minimizing weight loss and avoiding the need for enteral nutrition. Therefore, nutritional support with prophylactic insertion of a gastrostomy tube is frequently indicated, in order to maintain a satisfactory nutritional level and thus improve the nutritional status of the patient^(14)^.

## TECHNICAL ASPECTS

Percutaneous CT-guided gastrostomy should be carried out by an interventional radiologist with training in percutaneous procedures. Patients referred for the procedure are normally under the care of clinicians or surgeons, who, at some point decided to request the procedure, mainly after the failure of the endoscopic approach.

### Preparation

Patient assessment prior to the procedure is as important as the execution of a safe technique. In this context, certain aspects need to be taken into consideration: the overall health status of the patient; the level of consciousness; coagulation parameters; and the analysis of previous examinations to detect situations that could require additional measures, especially the presence of hepatomegaly or sequelae that would prevent the patient from remaining in a supine position. In addition, the patient and family should be informed as to the characteristics and inherent risks of the procedure^(15)^.

To minimize bleeding, oral anticoagulants should be stopped five days prior to the procedure, although any strategy needs to be adopted in consultation with the attending physician. The coagulation profile should be determined within the week prior to the procedure for patients under suspicion of coagulopathy, or even closer to the procedure for patients who have discontinued their anticoagulant medication. A platelet count < 50,000/mL is considered a relative contraindication, as are prothrombin and activated partial thromboplastin times > 1.5 above the standard. In such cases, a cost-benefit analysis should be carried out^(16,17)^.

A nasogastric tube should be inserted one day before the procedure, in order to fill the stomach with air and facilitate access, and patients should fast for at least six hours prior to the procedure. A gastropexy kit should be available to fix the gastric wall to the abdominal wall.

### Procedure

**Figure 1:**
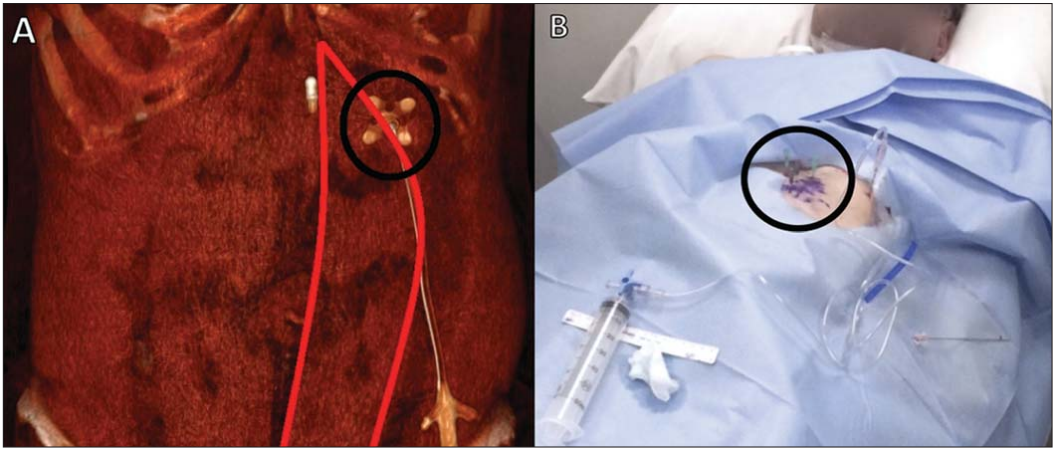
Marking the puncture site for CT-guided percutaneous gastrostomy. Three-dimensional CT reconstruction shows the appropriate site for the puncture, at the anterior abdominal wall, lateral to the rectus abdominis muscle (circle in **A**). It is necessary to mark three equidistant points, 3–4 cm apart, forming a triangle (circle in **B**).

**Figure 6:**
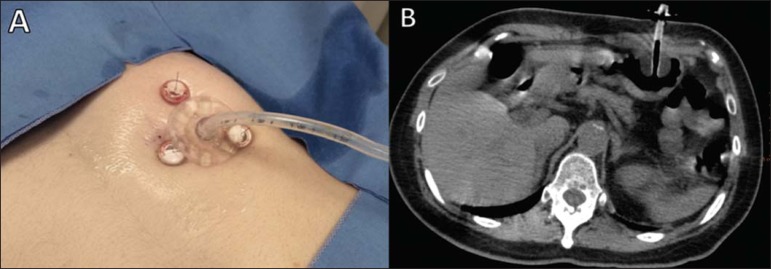
Final step of CT-guided percutaneous gastrostomy. After the correct positioning of the tube has been confirmed, it is fixed to the skin (**A**), and a follow-up CT is performed after gastric deflation (**B**)

Figures 1 to 6 illustrate CT-guided gastrostomy procedures, and the step-by-step technique is described below.

1. *Positioning the patient, monitoring, and sedation* – The patient should be placed in a supine position, and the vital signs should be monitored. Sedation, with fentanyl and midazolam, is initiated while the patient is conscious.
2. *Acquisition of images and choice of access* – The following steps are taken: injection of 1000 mL of room air for dilation of the gastric lumen; positioning of a metallic marker for orientation; and acquisition of axial tomography slices at the anterior abdominal wall, followed by the selection and marking of the puncture site with three equidistant points, 3–4 cm apart, in a triangle formation, the center of that formation corresponding to the introduction point for the gastrostomy tube, ideally on the wall anterior to the lower third of the gastric body, lateral to the rectus abdominis muscle, in order to avoid the epigastric arteries (Figure 1).
3. Scrubs, local asepsis, and sterile field placement.
4. *Local anesthesia* – Anesthesia of the entry point on the skin is administered via the peritoneum and gastric wall.
5. *Fixation of the gastric wall (gastropexy)* – The suture anchors (T-fasteners) are introduced by needle cannula at the three previously marked points of the triangle. After the correct positioning has been confirmed, the T portion of the fastener is ejected from the needle tip, the needle is withdrawn, the fastener is pulled tight with minimal pressure and held in place with crimps and a pledget (Figure 2).
  **Figure 2:**
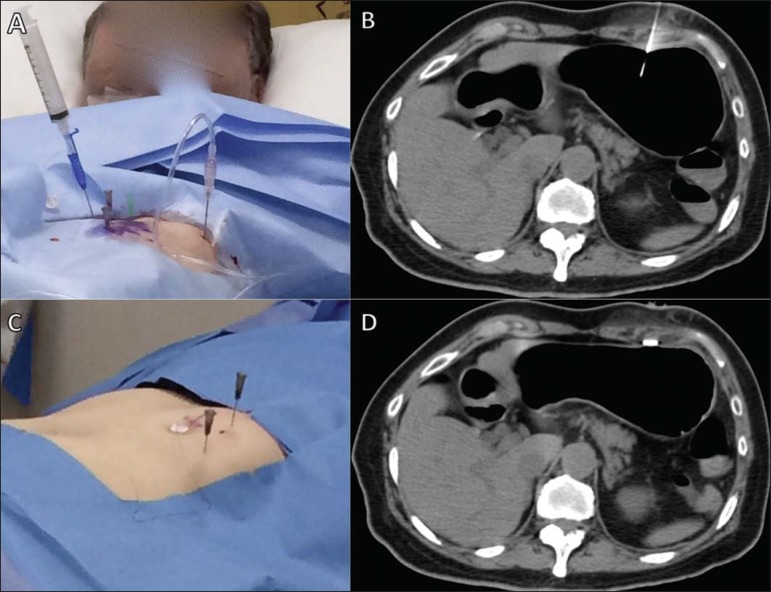
Fixation of the gastric wall for CT-guided percutaneous gastrostomy. Introduction of the needle cannula at one of the points of the previously marked triangle (**A**). After the correct positioning has been confirmed (**B**), the gastric wall is fixed at this point (**C,D**). The same procedure is repeated at the two other points of the triangle.
6. *Puncture, dilation, and passage of the gastrostomy tube* – At the center of the triangle demarcated by the pledgets, a small (≤ 1 cm) incision is made and transfixed with the puncture needle (Figure 3). The Seldinger technique is used in order to pass a pre-curved guidewire through the puncture needle, then withdraw the needle and introduce the dilator (Figure 4). Five successively larger dilator sleeves are introduced, after which the trocar is inserted and advanced. The system is then dismantled and the dilators are withdrawn. A small opening is created at the extremity nearest to the trocar, and the gastrostomy tube is passed through that orifice, the tissue being spread sufficiently to allow the tube to be inserted and advanced (Figure 5). After the tube has been introduced, the trocar is withdrawn.
  **Figure 3:**
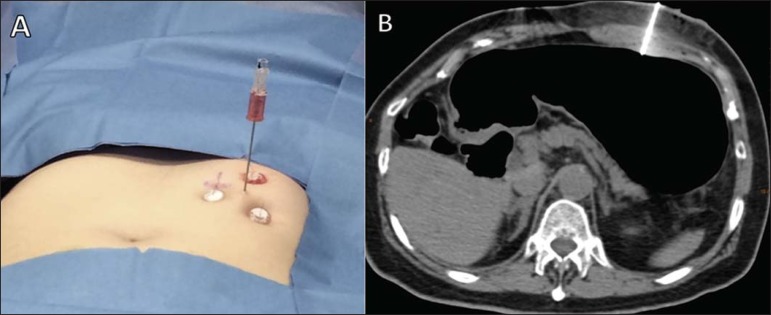
Gastric puncture for CT-guided percutaneous gastrostomy. A puncture needle is placed in the center of the previously established triangle (**A**) and the stomach is transfixed (**B**).
  **Figure 4:**
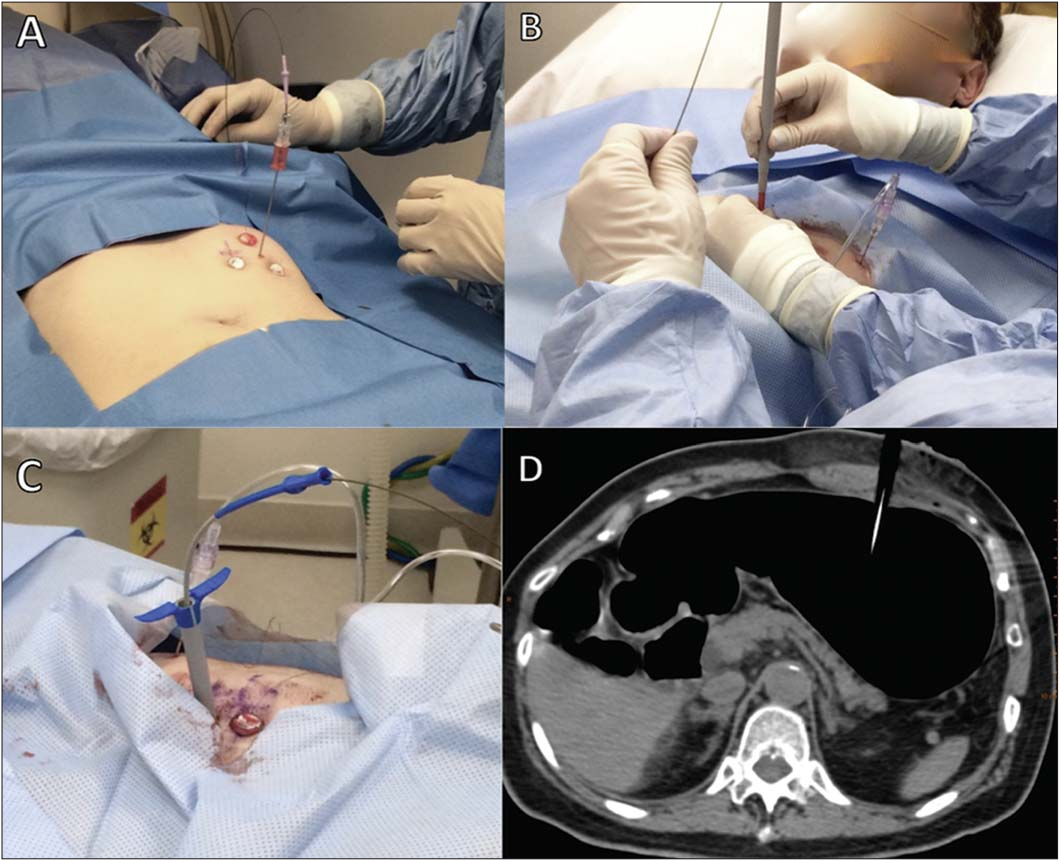
Passage of the guidewire and dilators for CT-guided percutaneous gastrostomy. The guidewire is passed through the interior of the gastric puncture needle (**A**), after which the needle is withdrawn and the guidewire is used in order to introduce the dilators and the trocar (**B,C**), as shown on the CT scan (**D**).
  **Figure 5:**
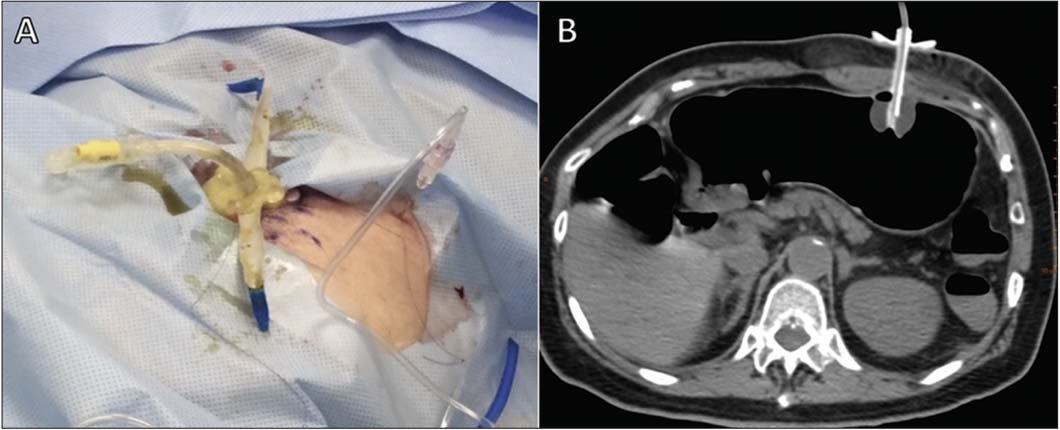
Introduction of the tube for the CT-guided percutaneous gastrostomy. The gastrostomy tube is passed through the trocar (**A**), and the balloon is inflated within the stomach (**B**).
7. *Fixing of the tube and finalization of the procedure (Figure 6)* – The following steps are taken: the balloon of the tube is inflated using 7–10 mL of distilled water; the tube is pulled and the plastic fixation device is advanced; the device is fixed with a 2-0 nylon suture; the area is cleaned and the sterile fields are removed; after the conclusion of the procedure, new images are obtained in order to identify immediate complications, such as massive pneumoperitoneum and bleeding or perforation of the liver or colon. It should be borne in mind that a small pneumoperitoneum is to be expected. In addition, the patient should be monitored until having completely recovered from the sedation.

### Post-procedure protocol

The stitches in the skin should be kept clean, kept dry, and monitored to detect any signs of infection. At two to three weeks after the procedure, the pathway between the abdominal wall and the gastric wall will be mature. That is the ideal moment to cut the threads of the T suture.

There is no consensus regarding the ideal time to initiate feeding through the recently implanted gastrostomy tube. Some authors recommend waiting 8 h, whereas others prefer to wait 48 h, in order to reduce the risk of extravasation.

## COMPLICATIONS

Complications related to gastrostomy are divided into three groups^(18)^: major complications; minor complications; and complications related to the tube. The major complications include peritonitis, external extravasation, massive hemorrhage, and bronchial aspiration. The indices of major complications in radiological techniques are relatively small, ranging from 1.4% to 5.9%, significantly lower than those for endoscopic techniques, which have been reported to be as high as 9.4%^(18,19)^. The minor complications include abdominal pain, peritoneal irritation, dehiscence of the incision, displacement or fracture of the catheter, gastroparesis and pneumonia. The rates of minor complications are similar among percutaneous techniques, ranging from 5.9% to 7.8%^(20)^.

Complications related to the tube are the most prevalent, being represented by occlusion or disintegration of the catheter or kinking of the tube. The rates of such complications are lower for radiological procedures than for endoscopic procedures (12% vs. 16%)^(18-20)^.

## CONCLUSION

CT-guided gastrostomy is a safe interventional radiological procedure, with a low risk of complication. Despite being relatively new in Brazil, the technique is increasingly more in demand in the country, mainly at centers that specialize in the treatment of head and neck tumors.
